# Roles of dual specificity tyrosine-phosphorylation-regulated kinase 2 in nervous system development and disease

**DOI:** 10.3389/fnins.2022.994256

**Published:** 2022-09-08

**Authors:** Gabriel Nicolás Santos-Durán, Antón Barreiro-Iglesias

**Affiliations:** Department of Functional Biology, Faculty of Biology, CIBUS, Universidade de Santiago de Compostela, Santiago de Compostela, Spain

**Keywords:** DYRK2, nervous system, disease, growth cone, spinal cord injury, cancer, epilepsy, neuroinflammation

## Abstract

Dual specificity tyrosine-phosphorylation-regulated kinases (DYRKs) are a group of conserved eukaryotic kinases phosphorylating tyrosine, serine, and threonine residues. The human DYRK family comprises 5 members (DYRK1A, DYRK1B, DYRK2, DYRK3, and DYRK4). The different DYRKs have been implicated in neurological diseases, cancer, and virus infection. Specifically, DYRK2 has been mainly implicated in cancer progression. However, its role in healthy and pathological nervous system function has been overlooked. In this context, we review current available data on DYRK2 in the nervous system, where the available studies indicate that it has key roles in neuronal development and function. DYRK2 regulates neuronal morphogenesis (e.g., axon growth and branching) by phosphorylating cytoskeletal elements (e.g., doublecortin). Comparative data reveals that it is involved in the development of olfactory and visual systems, the spinal cord and possibly the cortex. DYRK2 also participates in processes such as olfaction, vision and, learning. However, DYRK2 could be involved in other brain functions since available expression data shows that it is expressed across the whole brain. High DYRK2 protein levels have been detected in basal ganglia and cerebellum. In adult nervous system, DYRK2 mRNA expression is highest in the cortex, hippocampus, and retina. Regarding nervous system disease, DYRK2 has been implicated in neuroblastoma, glioma, epilepsy, neuroinflammation, Alzheimer’s disease, Parkinson’s disease, spinal cord injury and virus infection. DYRK2 upregulation usually has a negative impact in cancer-related conditions and a positive impact in non-malignant conditions. Its role in axon growth makes DYRK2 as a promising target for spinal cord or brain injury and regeneration.

## Introduction

Reversible phosphorylation is a key process regulating all aspects of cell biology like signal transduction, localization or half-life among other processes. Phosphorylation is carried out by protein kinases transferring phosphate groups from ATP (or GTP) to the hydroxyl groups of serine/threonine, or tyrosine residues of substrate proteins (Hanks and Hunter, 1995). The eukaryotic protein kinase superfamily (comprising Animalia, Plantae, Fungi, and Protista kingdoms) is organized in smaller subfamilies based on sequence homology within the kinase domain. “Dual-specificity Yak-related kinases” (DYRKs) are serine/threonine kinases related to the Yak protein from *Saccharomyces cerevisae*. DYRK also stands for “dual-specificity tyrosine-related kinase” since they auto-phosphorylate a tyrosine in their activation loop (Kentrup et al., 1996; Becker et al., 1998; Becker and Joost, 1999) though they should not be considered proper tyrosine kinases. DYRKs are further subdivided into two classes (human class I: DYRK1A, B; human class II: DYRK2, DYRK3, and DYRK4) according to sequences outside the kinase domain. These sequences determine additional biological properties like cellular localization, tissue distribution, or substrate specificity (Becker et al., 1998; Becker and Joost, 1999).

Mice defective for DYRK1A (Fotaki et al., 2002) and DYRK2 (Yoshida et al., 2020; Yogosawa et al., 2021) die at mid-gestation or birth, while null DYRK1B (Zhuang et al., 2022), DYRK3 (Sacher et al., 2007) or DYRK4 (Bogacheva et al., 2008) rodents are viable and fertile. Regarding protein and mRNA distribution in human tissues, the Human Protein Atlas^1^ found DYRK1A and DYRK2 in nearly all tissues examined (protein: 45 tissues; mRNA: 54 tissues). DYRK1B protein is detected in 2/3 of the tissues examined while DYRK1B mRNA has only relevant peaks on skeletal muscle and testis. There are no available data for DYRK3 and DYRK4 protein levels though both present high mRNA peaks in testis. Although all DYRKs have been involved in different diseases, including congenital malformations, cancer, and virus infection (Boni et al., 2020; Lindberg and Meijer, 2021; Yoshida and Yoshida, 2022), different lines of evidence suggest that DYRK1A and DYRK2 are the most relevant forms for human disease. DYRK1A is a well-known player in nervous system-related diseases and cancer (reviewed in Boni et al., 2020; Demuro et al., 2021; Lindberg and Meijer, 2021) while DYRK2 has been mainly implicated in cancer (reviewed in Yoshida and Yoshida, 2019, 2022; Correa-Sáez et al., 2020; Lindberg and Meijer, 2021; Tandon et al., 2021). However, the potential role of DYRK2 in nervous system-related conditions has been overlooked. Below we review the available current literature on DYRK2 with regards (i) neuronal morphogenesis; (ii) nervous system development and (iii) nervous system disease. General details on DYRK2 biology (chromosomic localization, sequence structure, cell functions, substrates, interactome, etc.) are beyond the scope of this mini-review and have been already covered in other works (see above).

## Methodology

For this mini-review we performed literature searches in Pubmed and Pubmed Central combining the keyword “DYRK2” with the following keywords: central nervous system, regeneration, spinal cord, spinal cord injury, spinal cord injury regeneration, degeneration, neurodegeneration, inflammation, aging, nervous system, brain, neuron, axon, axon growth, synapsis, neural connectivity, synaptic transmission, exogenesis, neurogenesis, neuronal differentiation, gliogenesis, axon elongation, neural crest, neuronal migration, growth cone, neuronal stem cells, stem cells, embryonic development, brain development, central nervous system development, spinal cord development, neuronal development, neuronal apoptosis, ciliopathies, ciliogenesis, peripheral nervous system, primary cilium, neurological diseases, neurological disorder, Parkinson, Parkinson’s disease, Alzheimer, Alzheimer’s disease, glioma, neuroblastoma, zebrafish, brain expression, spinal cord expression, central nervous system expression, RNA-seq, single-cell, ISH, hypoxia, ischemia, and stroke. When results retrieved exceeded 200 items only articles published in the last 5 years were evaluated for inclusion in the review article. We also performed searches for human DYRKs in the Human Protein Atlas (see text footnote 1) and DYRK2 in different atlases from the Allen Brain Map.^2^

## DYRK2 in neuronal morphogenesis

DYRK2 regulates growth cone dynamics for proper neuronal migration and branching *in vitro* and *in vivo*. Defects on these processes are associated with neurodevelopmental disorders (Prem et al., 2020). DYRK2 phosphorylates nuclear distribution element like-1 (NDEL1), which promotes actin dynamics leading to axon and dendrite outgrowth (Woo et al., 2019). The mechanism by which NDEL1 enhances actin-dynamics is not yet understood. However, it is known that NDEL1 phosphorylation requires the complexation of DYRK2, GSK3B, and TARA, an actin-associated protein, in the growth cones (Woo et al., 2019).

DYRK2 also phosphorylates doublecortin (DCX), a microtubule-associated protein (MAP), that constrains growth cone dynamics by stabilizing microtubules (Slepak et al., 2012). Interestingly, *in vitro* DCX or/and DYRK2 overexpression in rat hippocampal neurons have different effects. Compared to controls, DCX overexpression increases dendrite length but not branching, producing a “curvy” phenotype, while it does not affect axon length but reduces its branching. DYRK2 overexpression, decreases dendritic and axon length and branching. Strikingly, simultaneous DYRK2 and DCX overexpression, leads to a phenotype similar to that of controls by restricting dendrite length and branching while increasing axon length and branching (Slepak et al., 2012). This suggests a key DYRK2-DCX interplay for axon/dendrite development The authors suggest that DYRK2 controls DCX subcellular distribution and that a balanced ratio of phosphorylated DCX is required for proper arborization (Slepak et al., 2012). This work also revealed that overexpressing different DYRKs has different-even opposed-effects on axon and dendrite morphology.

DYRK2 is also involved in the phosphorylation of collapsin response mediator proteins (CRMPs), a family of neuron-enriched proteins that also regulate neurite outgrowth and growth cone dynamics (Cole et al., 2006). CRMPs, also known as dihydropyrimidinase-like proteins (DPYSLs), are mediator proteins of semaphorins (Morimura et al., 2013). DYRK2 phosphorylates CRMP2 and CRMP4, also known as Dpysl2 and Dpysl3, respectively, for proper positioning of spinal cord and neural crest cells *in vivo* (Tanaka et al., 2012; Morimura et al., 2013).

DYRK2 has been associated with the primary cilium, another cytoskeleton-related structure, involved in sensing the cellular environment (Gonçalves et al., 2021; reviewed in Singh and Lauth, 2017). Knocking down DYRK2 in mice prevents proper primary cilium development leading to an array of morphological defects (Yoshida et al., 2020; Yogosawa et al., 2021). Although these authors did not describe gross neural malformations, improper neuronal migration or branching were not assessed. The lack of neural tube defects would be surprising since the primary cilium mediates Shh signaling (Yogosawa et al., 2021), a key pathway for brain development (reviewed in Li et al., 2021).

DYRK2 has been also shown to phosphorylate nuclear factor of activated T cells (NFAT) in Drosophila cell lines (Gwack et al., 2006). Affected neuron branching was not analyzed though cannot be discarded since NFAT seems to have a key role in axon growth and guidance in vertebrate development (Nguyen and Di Giovanni, 2008). Finally, DYRK2 has been involved in neural specification of human pluripotent stem cells, though its role remains unclear (Bellmaine et al., 2017). Together, these data suggest that DYRK2 has a key role in neuron development, morphogenesis, dendrite/axon growth and neuronal migration and mainly through the modulation of cytoskeletal elements.

## DYRK2 in nervous system development

The distribution of DYRK2 across the nervous system of invertebrates and vertebrates is poorly understood. In the fly, DmDYRK2 is expressed in the third antennal segment and in the morphogenetic furrow of the eye corresponding to the boundary between proliferation and differentiation (Luebbering et al., 2013). Knocking down DmDYRK2 leads to impaired smell, impaired phototransduction, and subtle morphological defects in the eye. Ectopic DmDYRK2 expression in the eye causes gross morphological defects characterized by increased secondary, tertiary, and bristle interommatidial cells.

In zebrafish, DYRK2 has not been described in neural tissues before 12h post-fertilization stages (Sun et al., 2010, Tanaka et al., 2012; Morimura et al., 2013; Sun W. et al., 2017). At this and later stages, DYRK2 is ubiquitously expressed in the central nervous system being weakly expressed in the lateral spinal cord (Tanaka et al., 2012; Morimura et al., 2013). These works assessed DYRK2 loss of function leading to improper CRMP2 and CRMP4 phosphorylation. As result, some spinal cord cell types (Rohon-Beard interneuron misplacement: Tanaka et al., 2012; caudal primary motoneuron: Morimura et al., 2013) and neural crest cells (Tanaka et al., 2012) become misplaced.

Regarding rodents, DYRK2 deficient mice were generated in two different studies (Yoshida et al., 2020; Yogosawa et al., 2021). Neither of these two studies assessed DYRK2 expression in neural tissues. However, they describe aberrant Shh signaling together with congenital malformations and early pup death. Gross malformations concerned skeletal and lung development but not the neural tube. However, detailed analyses on neuronal migration or branching were not assessed and cannot be discarded (see above) due to the important role of Shh in nervous system development. To date, two reports confirmed DYRK2 expression in the central nervous system of mice by means of microarrays and ISH: in the cerebral cortex and hippocampus from E12.5 to 17.5 (Kudo et al., 2007); and in the cerebral cortex from E14.5 to adults (Diez-Roux et al., 2011).

Despite the scarce literature on neural DYRK2 expression/distribution, there are several resources where gene or protein detection can be browsed. The Allen Brain Map (see text footnote 2) has *in situ* hybridization (ISH), microarray and/or RNA-seq expression data organized according to different species, stages of development, and brain structures. In developing mice, currently there are no DYRK2 data.^3^ In 56 postanal days-old mice (P56, considered adult mice) DYRK2 expression (ISH) is detected in the isocortex and hippocampus at medium levels^4^ but not in the spinal cord,^5^ which is in agreement with previous reports (Kudo et al., 2007; Diez-Roux et al., 2011). Therefore, in the murine nervous system DYRK2 is expressed in the isocortex and hippocampus, but not in other structures, from E12.5 to adult stages.

In developing human brains, DYRK2 has been detected by means of RNA-seq.^6^ This browser retrieves data restricted to the cortex, but not other brain structures, from 8 weeks post-conception to 40 years old. For all the cortical regions considered, DYRK2 is expressed from 8 to 24 weeks post-conception (mid-gestation). Since then, it is downregulated and not expressed in adults, suggesting it is involved in proliferation and/or early differentiation of the cortex. For the whole brain of adult humans (20–60 years old), there are laser microdissection microarray data on DYRK2 expression.^7^ In agreement with data on the developing brain, DYRK2 is absent from cortical regions at the analyzed ages. However, DYRK2 seems to be expressed in most non-cortical regions of adult humans suggesting a role for DYRK2 in constitutive neuronal functions of other brain regions.

**FIGURE 1:**
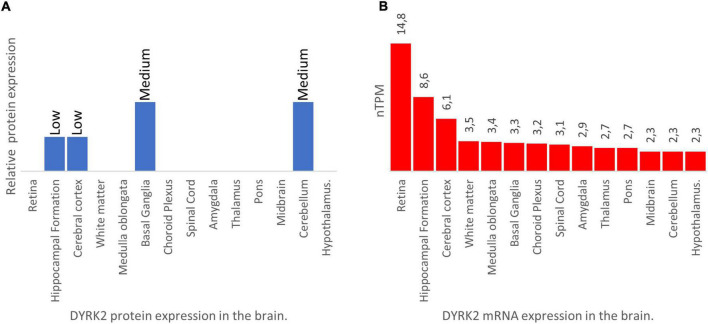
DYRK2 mRNA and protein brain distribution according to the Human Protein Atlas (https://www.proteinatlas.org/). **(A)** DYRK2 protein data from fluorescent immunohistochemistry experiments. The data corresponds to the “protein expression dataset.” Units represent relative fluorescence to the maximum fluorescence found across all the tissues probed (neural and non-neural). **(B)** DYRK2 mRNA data from RNA-seq experiments. The data corresponds to the “consensus dataset.” Units are expressed as number of transcripts per million (nTPM).

The Human Protein Atlas (see text footnote 1) is another source where protein and RNA high-throughput data can be browsed (Thul and Lindskog, 2018). This database contains information from adult human samples (18–94 years old) but also adult pig and mouse samples. Search output also retrieves RNA-Seq data from the Genotype-Tissue Expression consortium (Ardlie et al., 2015) and CAGE data from the FANTOM5 consortium (Yu et al., 2015). The amount of DYRK2 protein in the brain is comparable to that of other tissues (low to medium levels; Figure 1A). It is detected in the 4 brain regions examined: the cortex and hippocampus have low protein levels whereas the cerebellum and putamen present medium protein levels. Regarding DYRK2 mRNA, according to the consensus data set, which normalizes data from the Human Protein Atlas, Genotype-Tissue Expression and FANTOM5, the brain presents the lowest expression levels compared with other tissues. Within the brain, DYRK2 mRNA is homogenously expressed in the 14 brain structures analyzed, though presenting higher peaks in the retina, hippocampus, and cortex (Figure 1B). Strikingly, there seems to be a disagreement between Allen Brain Map and the Human Protein Atlas with regards to the cortex since the latter detected DYRK2 expression while the former did not. This is probably due to the different sensibility of the techniques used to obtain the expression data (microarrays vs RNA-seq and immunohistochemistry). RNA-seq data from pig and mouse shows similar DYRK2 expression profiles being highest in retina, hippocampus and cortex and rather homogenous in other brain structures.

With regards to normal/constitutive brain function, there is even less data on DYRK2, but Katzman et al. (2021) have shown transient DYRK2 upregulation in the hippocampus associated to metabolic increase following avoidance learning.

To summarize, in the context of nervous system ontogeny, DYRK2 seems to be involved in the development of the retina, olfactory system, spinal cord, and cortex in different invertebrate and vertebrate species. Besides, it seems to have functions in olfaction, phototransduction, and learning and might carry out constitutive cellular functions across the whole brain. More research is needed to analyze the role of DYRK2 in the different brain regions showing mRNA or protein expression.

## DYRK2 in nervous system diseases

DYRK2 misfunction has been mainly associated with cancer (reviewed in Correa-Sáez et al., 2020 and Tandon et al., 2021) and other conditions affecting the nervous system. Regarding nervous system malignancies, Uhl found DYRK2 and DYRK3 upregulation in neuroblastoma (an aggressive tumor affecting the sympathetic system and/or adrenal glands in early childhood) patients with poor prognoses (Uhl et al., 2018). Besides, treating neuroblastoma cell lines with LDN-192960, a DYRK2 inhibitor, resulted in a high cytotoxic effect suggesting a key role for DYRK2 in the survival of tumoral cells (Uhl et al., 2018). Park also reported that an *in vitro* drug treatment that inhibited neuroblastoma growth involved DYRK2 downregulation (Park et al., 2002). All together these data reveal that DYRK2 might promote the survival and growth of neuroblastoma cells.

DYRK2 is also associated to gliomas, the most general primary nervous system tumor, which is characterized by a forceful malignant proliferation and invasion and high mortality. Bidinotto et al. reported DYRK2 upregulation in Brazilian glioblastomas (Bidinotto et al., 2016). In two murine models of glioma, Wang et al. also reported upregulation of 41 kinases including DYRK2 (Wang et al., 2019). These models were generated with p53-null astrocytes modified with PDGFRA or NTRK1 oncogenes that were injected into athymic nude mice. On the contrary, Shen et al. reported that DYRK2 downregulation correlates with low patient survival, and higher malignancy degrees (Shen et al., 2017). Besides, in human glioma cell lines, DYRK2 downregulation resulted in migration up-regulation and lower E-cadherin expression both associated with cancer progression and metastasis (Shen et al., 2017). Interestingly, DYRK2 overexpression resulted in migration inhibition *in vitro* and also decreased phosphoinositide 3-kinase (PI3K), protein kinase B (AKT), Glycogen Synthase Kinase 3β (GSK3β) protein complex levels, which are associated with epithelial-mesenchymal transition inhibition (Zhou et al., 2015). These contradictory results suggest that the role of DYRK2 in gliomas might be cell-, tissue- or patient-specific.

DYRK2 has been related to epilepsy, a neurological condition not well understood and characterized by recurrent seizures (Yuen et al., 2018). Yu et al. described a type of epilepsy (temporal lobe epilepsy) characterized by hippocampal sclerosis, which causes neuronal apoptosis in the hippocampus (Yu et al., 2021). These authors showed how the aberrant expression of certain non-coding RNAs leads to DYRK2 downregulation triggering neuronal apoptosis in human patients. Haenisch et al. revealed that miRNA-187-3p is up-regulated in rat models of epilepsy and that it targets DYRK2 and potassium channel KCNK10/TREK-2 mRNAs (Haenisch et al., 2016). Strikingly, changes in miRNA-187-3p expression correlate with KCNK10/TREK-2 but not with DYRK2 protein levels.

DYRK2 has been also involved in neuroinflammation processes in which activated microglia and astrocytes release cytokines, like tumor necrosis factor-alpha (TNF-α) and interleukin-1β (IL-1β), promoting neuronal damage. In human cell line models, Xu et al. showed, by means of immunofluorescence and immunoblotting, that lipopolysaccharide (LPS) activated microglia upregulate DYRK2, phosphorylated protein 65 (pP65), and phosphorylated mitogen-activated kinase protein 38 (pMAPK38), leading to cytokine release (Xu et al., 2018). Strikingly, they also show that DYRK2 overexpression has a protective effect since it downregulates phosphoinositide3-kinase/Akt, which is involved in the attenuation of LPS-induced responses. In neurons, Sun Y. et al. (2017), using the same methodological approaches, found that DYRK2 is involved in the activation of apoptotic pathways after LPS treatment. However, again DYRK2 overexpression has a protective effect inhibiting TNF-α release, which is involved in cell death, and neuronal apoptosis. Regarding animal models, in senescence accelerated mouse prone 8 (SAMP8) mice, which present depression-like behavior and hippocampal inflammation, there is DYRK2 downregulation compared to senescence accelerated mouse resistant 1 (SAMR1) mice (they are considered as normal aging controls) as demonstrated by RNA-seq experiments (Ito et al., 2022). Brandão et al., in a rodent inflammatory model associated with a high fat diet, also showed slight DYRK2 upregulation in RNA-sequencing experiments in the nucleus accumbens, which is a critical structure modulating chronic pain (Brandão et al., 2020).

Sparse studies also involved DYRK2 function in other pathologies associated with the nervous system. Immunoblotting experiments demonstrated that DYRK2 is also involved in the phosphorylation of the tau protein *in vitro* (Woods et al., 2001). This phosphorylation takes place in the same threonine that is phosphorylated in tau from newborn rats and hyper-phosphorylated in filamentous tau from Alzheimer’s disease brains (Morishima-Kawashima et al., 1995). Whether phosphorylation by DYRK2 in this residue causes pathology has not yet been investigated. In the same line, DYRK2 seems to modulate the oligomerization-aggregation of alpha-synuclein, a key hallmark of Parkinson’s Disease (Gonçalves et al., 2016; Gonçalves and Outeiro, 2017). Knocking down DYRK2 promotes oligomerization of alpha-synuclein suggesting a protective role for DYRK2 in Parkinson’s Disease since oligomers are considered toxic. Recent RNA-sequencing work in the lamprey model of spinal cord injury has shown that a baclofen treatment, which promotes neuronal survival and axonal regeneration in brainstem descending neurons, increases DYRK2 expression in the brainstem (Sobrido-Cameán et al., 2020), which suggests a positive role for DYRK2 in neuronal survival and/or regeneration after spinal cord injury. Regarding virus-related pathologies, Chailangkarn et al., showed, by means of nano-liquid chromatography and tandem mass-spectrometry, that DYRK2 is involved in the pathogenesis of Rabies virus since it becomes downregulated in pluripotent stem cell neurons after infection (Chailangkarn et al., 2021). To summarize, a growing body of evidence suggests that DYRK2 is mainly involved in neuroblastoma, glioma, epilepsy, and neuroinflammation but also could be involved in traumatic and neurodegenerative diseases and Rabies virus infection. Despite the lack of a general rule, we can sketch two trends regarding the activity of DYRK2: in cancer-related it has a general negative impact, whereas in non-cancer-related contexts it has a general positive impact.

## Conclusion

DYRK1A and DYRK2 are the most relevant DYRK forms and well-known players in cancer progression (Correa-Sáez et al., 2020; Demuro et al., 2021; Yoshida and Yoshida, 2022). However, the potential role of DYRK2 in brain function and disease has been overlooked. In the present work, we reviewed current data on DYRK2 function with regards to neuronal morphogenesis, nervous system development, and nervous system disease. DYRK2 is a key player regulating the cytoskeleton and thus neuronal migration and neurite outgrowth and branching. *In vivo* studies suggest that DYRK2 could have a role in the correct placement of neurons across the neural tube. Human expression data suggest that DYRK2 could have a specialized role in cortical development but also in constitutive functions across the whole brain. Comparative data suggest functions in photoreception, olfaction, and memory. There is also a growing body of evidence suggesting the involvement of DYRK2 in the normal and pathological function of the nervous system. From a therapeutic perspective, this review points DYRK2 as a key player regulating cytoskeletal dynamics and axon growth placing this protein as a promising target for spinal cord and brain injury and regeneration that awaits future *in vivo* research.

## Author contributions

AB-I defined the scope, the article format, main points to be covered, and edited the manuscript. GS-D performed the database search and the manuscript writing. Both authors contributed to the article and approved the submitted version.

## Footnotes

1
https://www.proteinatlas.org/

2
https://portal.brain-map.org/

3
https://developingmouse.brain-map.org/

4
https://mouse.brain-map.org/

5
https://mousespinal.brain-map.org/

6
https://www.brainspan.org/

7
https://human.brain-map.org/
